# The Race to Salvage Glucocerebrosidase: Understanding Small‐Molecule Therapies for *GBA1* ‐Associated Parkinsonism

**DOI:** 10.1002/mds.70168

**Published:** 2025-12-29

**Authors:** Mark J. Henderson, Tiffany C. Chen, Logan M. Glasstetter, Yu Chen, Juan J. Marugan, Ellen Sidransky

**Affiliations:** ^1^ Division of Preclinical Innovation National Center for Advancing Translational Sciences, National Institutes of Health Rockville Maryland USA; ^2^ Medical Genetics Branch National Human Genome Research Institute, National Institutes of Health Bethesda Maryland USA

**Keywords:** activators, chaperones, Gaucher disease, *GBA1*, glucocerebrosidase, Parkinson's disease

## Abstract

Variants in *GBA1*, the gene encoding the lysosomal enzyme glucocerebrosidase, cause Gaucher disease and confer an increased risk for parkinsonism. Strategies using small molecules can improve the function of glucocerebrosidase in lysosomes. A clear understanding of the mechanism‐of‐action of these compounds will facilitate development of *GBA1‐*modulating drugs for Parkinson's disease.

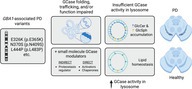

The discovery that variants in *GBA1*, the gene encoding the lysosomal enzyme glucocerebrosidase (GCase), confer an increased risk for parkinsonism^1^ generated great enthusiasm, as it presented a genetically verified and druggable target for the treatment of Parkinson's disease (PD). Individuals carrying a single variant in the *GBA1* gene (heterozygotes) and those with biallelic pathogenic *GBA1* variants resulting in Gaucher disease (GD), the inherited deficiency of GCase, share this increased PD risk. While the precise mechanistic underpinnings connecting GCase dysfunction and PD etiology have remained elusive, interest in GCase as a therapeutic target for PD has persisted.^2^ Directing GCase‐modifying therapeutics to the brain remains a major challenge, however, as current treatments for GD do not cross the blood–brain barrier.

Recent efforts have focused on developing brain‐penetrant small‐molecule drugs that target GCase. While different academic centers and pharma companies have active programs in this field, with a handful of compounds already in clinical trials, we have observed considerable confusion regarding this new line of therapeutics and what they seek to achieve. Some of these small molecules are inhibitory chaperones, others non‐inhibitory chaperones, and some are designed to be activators, but have very little or no chaperone capacity. Still other small‐molecule drugs target synthesis of the stored lipids to circumvent the loss of GCase (Fig. 1). Here we outline the various types of small molecules that target GCase and discuss how their mechanisms of action may impact their utility as drugs for PD.

**FIG. 1:**
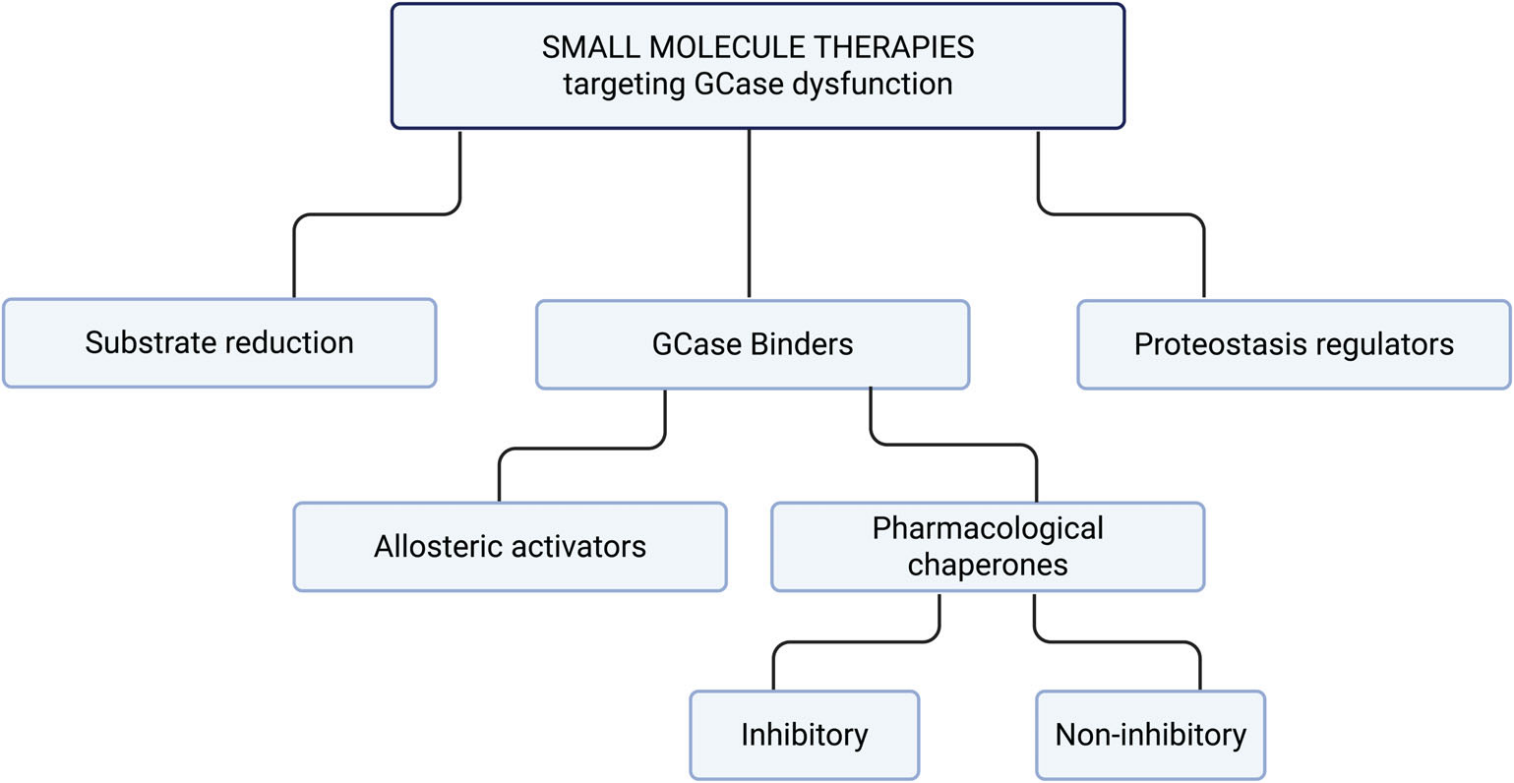
Different types of small‐molecule drugs targeting glucocerebrosidase (GCase)‐associated Parkinson's disease (PD). The flowchart differentiates between the types of GCase binders. Figure created in BioRender.

To understand the mechanistic differences between types of small‐molecule modulators of GCase, it is necessary to delineate their points of intervention (Fig. 2). After synthesis in the endoplasmic reticulum (ER), several structural modifications occur (eg, N‐glycosylation), and endogenous chaperones (eg, heat‐shock proteins) interact with GCase to facilitate its proper folding. Improperly folded GCase is re‐glycosylated, and folding is re‐attempted, but if proper folding repeatedly fails, it will be targeted for ER‐associated degradation (ERAD) and removed.^3^ Properly folded GCase is trafficked through the Golgi apparatus to the lysosome, where the enzyme associates with the inner face of the lysosomal membrane to become enzymatically active.^3^ If each of these steps are achieved, GCase can then interact with sphingolipid substrates to perform its critical enzymatic function, thereby maintaining lipid homeostasis.

**FIG. 2:**
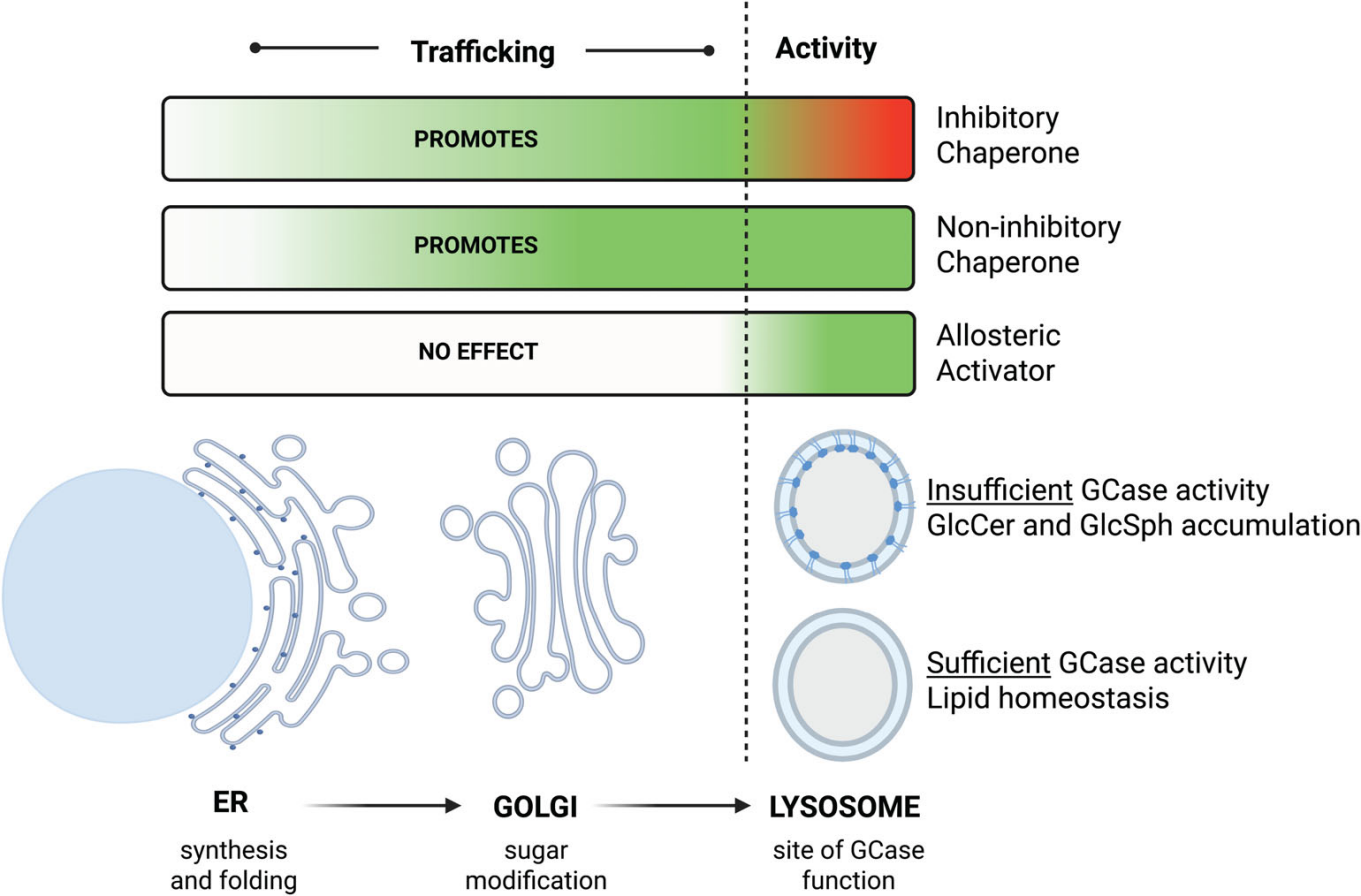
Glucocerebrosidase (GCase)‐targeting molecules will impact different stages of GCase transport and function. Correct synthesis and folding of GCase avoids endoplasmic reticulum (ER)‐associated degradation (ERAD) and enhances transit to the Golgi and lysosome, steps which are promoted by chaperones. Activators do not alter trafficking but increase activity of lysosomal GCase. Color scale indicates small‐molecule effect on GCase at the different stages of biogenesis and lysosomal function (red = decreased, green = increased). GlcCer, glucosylceramide; GlcSph, glucosylsphingosine. Figure created in BioRender.

Proteostasis regulators are one class of small‐molecule modulators for GCase, although they are indirect modifiers of the enzyme.^4^ These could conceivably be developed to target important cofactors of GCase impacting its delivery to the lysosome, such as LIMP‐2, or could behave as more broadly‐acting proteostasis regulators that promote GCase folding over degradation. One component of the GCase proteostasis network, FKBP10, accelerates the degradation of mutant GCase, so FKBP10‐interfering compounds could promote its folding and trafficking.^5^ Also, small molecules that modulate PIKfyve activity, coupled with an integrated stress response inhibitor, can improve cellular GCase activity.^6^ Recently, a CRISPR‐based study identified the Commander complex as another critical regulator of GCase trafficking and function.^7^

In contrast to the indirect modulators, small molecules that directly bind GCase, including enzyme activators and pharmacological chaperones, can more specifically alter the enzyme's function. The decision regarding which therapeutic mechanism would be most beneficial for a given patient is inherently dependent on understanding the nature of the mutated protein. Is it properly folded but with reduced enzymatic activity? Or does it not fold well and can never make its way out of the ER? (Fig. 2).

Since activators and pharmacological chaperones of GCase result in differing enzyme conformations with separable modes of action, their effects do not always correlate. This distinction between pharmacological actions of activators versus chaperones has also been recognized in the cystic fibrosis field, where correctors that improve folding (eg, lumacaftor) and potentiators that increase activity (eg, ivacaftor) have been developed to address loss‐of‐function *CFTR* mutations.^8^

Unfortunately, in the GCase and lysosomal enzyme literature, these two terms are often used interchangeably. It remains uncertain which pharmacological profile will be best suited for the correction of GCase insufficiency in PD. Nonetheless, the success of future clinical trials will depend on proper characterization of the mechanism of action for each compound, coupled with careful preclinical validation. Furthermore, with the continued emergence of new small molecules targeting GCase, it will be important to develop standardized evaluations to facilitate comparisons and to inform clinical trials.

Enzyme activators aim to increase the catalytic efficiency of protein expressed from the wildtype or mutant allele and therefore compensate for GCase haploinsufficiency. Mechanistically, they are allosteric binders that alter the active site conformation, improving the enzyme's affinity toward the transition state and reducing the activation energy of the reaction. Because the overall structure and conformation of the transition state of a reaction is substrate‐dependent, a molecule's capacity for improving the hydrolysis of fluorescent substrates, which are commonly used to characterize GCase enzyme activity, might not accurately predict activation towards natural substrates (glucosylceramide and/or glucosylsphingosine). Cleavage of the natural substrates can be measured using deuterated lipids, coupled with mass spectrometry (MS)‐based detection, but these assays are challenging to implement. Since the global conformation of an enzyme is sequence‐dependent, the potency and efficacy of an activator is also mutation‐dependent. We believe that the source of GCase should be cell lysates or tissue homogenates from patients with different *GBA1* genotypes like WT/WT, N370S/N370S (p.N409S/p.N409S), L444P/L444P (p.L483P/p.L483P), etc., in order to best reproduce the intrinsic physiological conditions relevant to GCase activation (eg, the presence of natural activators like saposin C and phosphatidylserine).^9^ Notably, even if enzymatically active in homogenates, some forms of mutated GCase are unable to get to the lysosome. For example, certain variants like L444P generate an unstable protein that is mostly removed by ERAD, trapped in the Golgi, and/or degraded within the lysosome.^3^, ^10^ An activator would not be very effective in this situation. Additionally, activators would not address the potential burdens caused by misfolded GCase expressed from the variant‐bearing allele, which may also contribute to PD pathogenesis.

Pharmacological chaperones, in contrast, are particularly relevant for *GBA1* variants, as they are designed to stabilize GCase, even when mutated. They act by inducing conformations that accelerate the kinetics of folding and increase the minimum energy conformation fraction at the ER. The folded enzyme is then glycosylated and transported in vesicles to Golgi, and eventually to the lysosome. Unlike enzyme activators, pharmacological chaperones can either bind to the active site of GCase as inhibitory chaperones, or allosterically as non‐inhibitory chaperones.^11^

The first series of GCase pharmacological chaperones described were active‐site inhibitors^12^ designed to be mimetics of the transition state of sphingolipid hydrolysis to efficiently translocate GCase to the lysosome. It was hoped that the affinity of these compounds toward GCase would be pH‐dependent, displaying greater binding at the ER to facilitate folding and translocation, yet reduced binding at the lower lysosomal pH, where the chaperone would dissociate from the enzyme and/or be displaced by accumulated substrate.^13^ Most of this work has focused on a handful of prototype iminosugar scaffolds,^14^, ^15^, ^16^ including *N*‐alkyl deoxynojirimycin (DNJ) and isofagomine.^17^, ^18^, ^19^ An initial high‐throughput screen for GCase chaperones, performed with recombinant GCase, uncovered several new classes of inhibitory chaperones.^20^ However, since inhibitory chaperones compete with the natural substrate, their window of therapeutic efficacy is limited – if the dose is too low, no effect will be seen, whereas if too high, it can inhibit the protein's function.^21^ In addition, they have poor selectivity against other lysosomal hydrolases and non‐lysosomal GBA isoforms.^14^

A screen of a library of 1040 U.S. Food and Drug Administration (FDA)‐approved drugs identified ambroxol, a compound used to treat airway mucus hypersecretion and hyaline membrane disease in newborns, as an enzyme enhancer able to increase GCase activity.^22^ Ambroxol is a mixed‐type inhibitor of GCase, with maximal inhibition at neutral pH and negligible inhibition at lysosomal pH. There are scattered reports of neurological improvements in patients with type 3 GD treated at high ambroxol doses^23^, and different clinical trials using ambroxol for GD and for *GBA1‐*associated PD or other Lewy body disorders are underway.^24^, ^25^, ^26^ In addition to its reported chaperone activity, ambroxol also has GCase‐independent mechanisms of action including effects on ERAD and autophagy.^27^

Given the liabilities of active‐site inhibitor chaperones, an alternate strategy is the development of non‐inhibitory, allosteric site‐directed chaperones, with a wider therapeutic window. The first were discovered through high‐throughput screening of 250,000 compounds utilizing spleen homogenate from a patient with GD and a fluorogenic substrate as a readout for N370S GCase activity.^9^ Two hits were advanced by medicinal chemistry efforts resulting in NCGC607 and NCGC758 (patented as US9464035B2 and US9353117B2, respectively). The NCGC758 series was out‐licensed to a pharmaceutical company and optimized as an allosteric enzyme activator, yielding BIA 28–6156, which is currently in a phase IIB clinical trial for *GBA1*‐associated PD (NCT05819359).^28^

The majority of the GCase chaperones reported in the literature have been identified and optimized by their ability to ‘activate’ GCase toward the hydrolysis of the fluorescent substrate 4‐methylumbelliferyl β‐D‐glucopyranoside in whole‐cell extracts. However, this criterion does not align with the true function of pharmacological chaperones, which are intended to salvage misfolded protein from ERAD within intact cells. Therefore, evaluating GCase stabilization in live cells is a more appropriate means to quantify the pharmacological chaperone capacity of allosteric binders. Since this is a conceptually different approach, new assay strategies are necessary.^29^ The development of non‐inhibitory chaperones has been hindered by the lack of robust and high‐throughput cell‐based assays to evaluate GCase stabilization, trafficking, and lysosomal activity.

Recently, we reported our lengthy odyssey to develop appropriate assays to validate, functionally dissect, and harmonize the discovery of activators and pharmacological chaperones of GCase.^29^ The pipeline (Fig. 3) consists of complementary cell‐based assays that measure GCase levels, trafficking,^30^ and lysosomal activity,^31^ with biophysical approaches confirming direct binding to GCase. These include microscale thermophoresis (MST) and surface plasmon resonance (SPR), which measure the binding affinity of the compound to GCase, as well as differential scanning fluorimetry (DSF) and cellular thermal shift assays (CETSA)^32^ to measure compound‐induced alterations in GCase thermal stability. Functional effects on enzyme activity are characterized using MS assays that directly measure glucosylceramides and glucosylsphingosine from cells. We believe that cell models exhibiting a lipid accumulation phenotype (eg, induced pluripotent stem cell [iPSC]‐derived macrophages from patients with GD) or PD‐relevant phenotypes (eg, iPSC‐derived dopaminergic neurons or microglia) should be used for the characterization of GCase non‐inhibitory chaperones. This collection of robust cell‐based methods can discriminate GCase activators from pharmacological chaperones, which is necessary for the proper evaluation and development of these molecules.

**FIG. 3:**
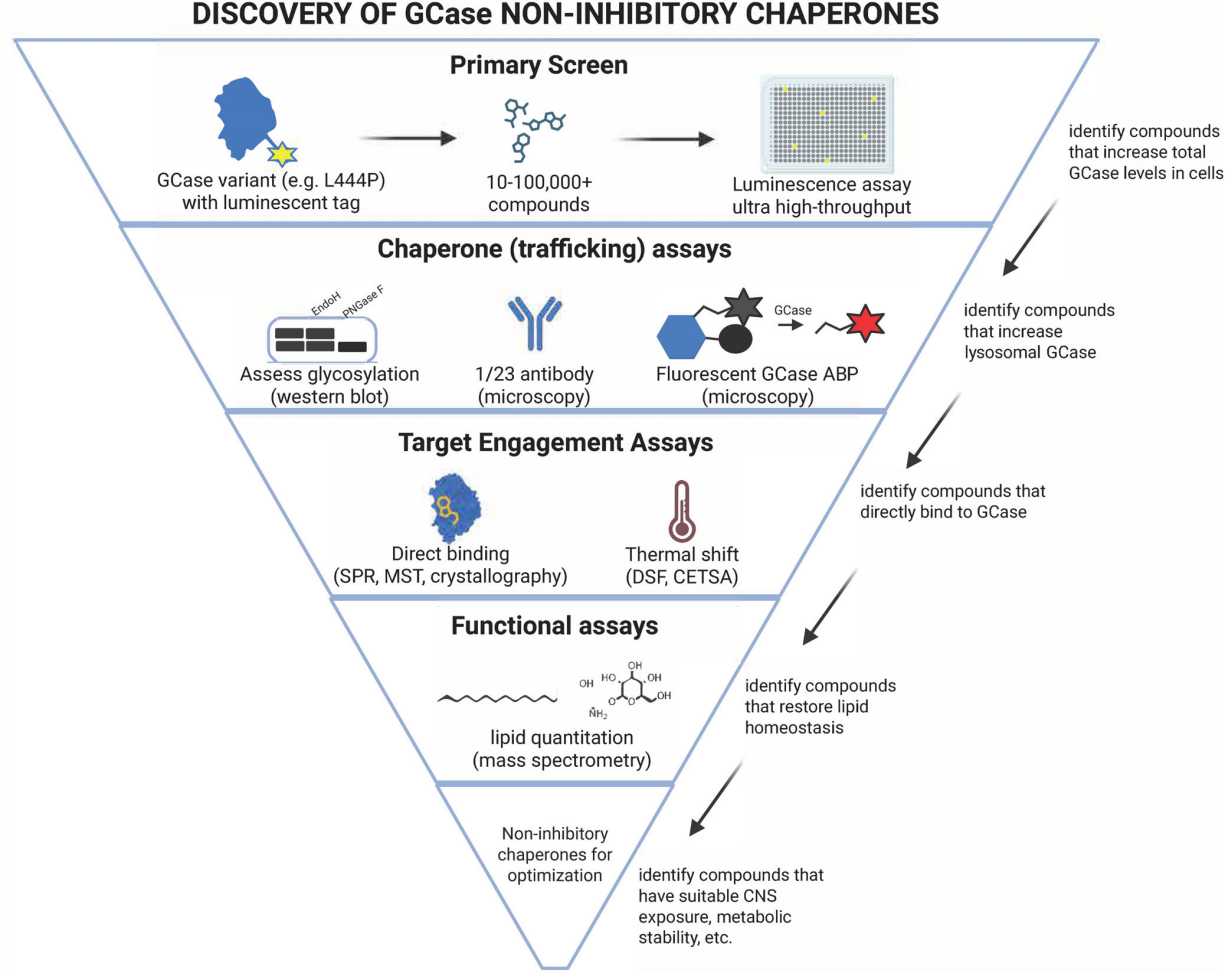
Pipeline for screening, validating, and developing small‐molecule lead non‐inhibitory chaperones of glucocerebrosidase (GCase). The pipeline includes a primary screen for compounds that increase total GCase in cells, followed by trafficking assays to establish which compounds actually increase lysosomal GCase. Target engagement assays are performed to identify compounds which directly bind to GCase, and lead compounds are then evaluated by functional assays to assess their ability to restore lipid homeostasis using mass spectrometry. The best candidates are optimized to improve stability, central nervous system (CNS) permeability, biodistribution, and so on. ABP, activity‐based probe; CETSA, cellular thermal shift assays; DSF, differential scanning fluorimetry; MST, microscale thermophoresis; SPR, surface plasmon resonance. Figure created in BioRender. [Color figure can be viewed at wileyonlinelibrary.com]

In recent years, there has been increasing evidence that glucocerebrosidase tends to dimerize in a concentration‐dependent fashion,^33^ confirmed by X‐ray crystallography. Many non‐inhibitory allosteric chaperones are thought to target the dimer interface,^33^, ^34^ facilitating the formation of GCase dimers and kinetically accelerating the folding process. Recently, a second allosteric site on GCase has been proposed.^11^ A challenge to the development of non‐inhibitory chaperones will likely be the delicate balance between optimizing binding to the ‘greasy’ pockets of currently discovered allosteric sites while maintaining brain penetrance and solubility.

Improving GCase function with small molecules is predicted to improve the lipid imbalance caused by enzyme dysfunction. In patients with GD, the lipid substrates glucosylceramide and glucosylsphingosine are both elevated. Glucosylceramide accumulates in lysosomes, particularly in macrophages. Notably, the mere reduction of glucosylceramide in individuals with *GBA1*‐related PD failed to improve their disease course, as demonstrated by Sanofi's multisite clinical trial (MOVES‐PD) of venglustat, a small‐molecule inhibitor of glucosylceramide synthase.^35^, ^36^ Venglustat‐treated patients showed a substantial reduction in glucosylceramide in plasma and cerebrospinal fluid (CSF) but also trended towards a worsening of PD symptoms, and the trial was terminated early. Thus, small‐molecule inhibitors of ceramide synthesis do not appear to be an appropriate therapeutic strategy for PD. A second lipid, glucosylsphingosine, although present at much lower concentrations than glucosylceramide, is often elevated by orders of magnitude and can be detected in plasma, CSF, and dried blood spots.^37^ Studies have indicated that glucosylsphingosine can promote aggregation of α‐synuclein in experimental models of *GBA1*‐associated PD.^38^ Carriers of *GBA1* variants, both with and without PD, show a very modest increase in plasma glucosylsphingosine.^39^ The possibility that an accumulation of glucosylsphingosine within the central nervous system contributes to *GBA1*‐associated PD requires further investigation.

These are indeed exciting times for the development of new therapeutic strategies that may impact the progressive course of PD. Clinical trials of *GBA1*‐modulating therapeutics will be challenging with regard to the optimal timing of the intervention and appropriate outcome parameters.^40^ But should these trials prove successful, this strategy has the potential to alter the progressive course of *GBA1*‐associated PD. Furthermore, this therapeutic approach may also have broader implications and impact for idiopathic PD, due to the known inverse relationship between GCase and α‐synuclein.^41^

## Author Roles

(1) Research Project: A. Conception, B. Literature Review; (2) Manuscript Preparation: A. Writing of the First Draft, B. Editing of Final Version.

M.J.H.: 1A, 2A, 2B.

T.C.C.: 1B, 2A, 2B.

L.M.G.: 1B, 2B.

Y.C.: 2B.

J.J.M.: 1A, 2B.

E.S.: 1A, 1B, 2A, 2B.

## Financial Disclosures of All Authors (for the Past 12 Months)

The Sidransky laboratory (Y.C., T.C.C., L.M.G., and E.S.) has received research funding from The Michael J. Fox Foundation, Roche, SPARK‐NS, and ASAP. M.J.H. and J.J.M. received research funding from The Michael J. Fox Foundation, Roche, and SPARK‐NS.

## Financial Disclosures and Conflicts of Interest

Author disclosures are available in the S1.

## Supporting information


**Data S1.** Coi_disclosure sidransky.

## Data Availability

Data sharing not applicable to this article as no datasets were generated or analysed during the current study.
